# Analysis of carotid artery deformation in different head and neck positions for maxillofacial catheter navigation in advanced oral cancer treatment

**DOI:** 10.1186/1475-925X-11-65

**Published:** 2012-09-04

**Authors:** Takashi Ohya, Toshinori Iwai, Kuan Luan, Takashi Kato, Hongen Liao, Etsuko Kobayashi, Kenji Mitsudo, Nobukazu Fuwa, Ryuji Kohno, Ichiro Sakuma, Iwai Tohnai

**Affiliations:** 1 Department of Oral and Maxillofacial Surgery, Yokohama City University Graduate School of Medicine, 3-9 Fukuura, Kanazawa-ku, Yokohama, 236-0004, Japan; 2 Graduate School of Engineering, The University of Tokyo, 7-3-1 Hongo, Bunkyo-ku, Tokyo, 113-8656, Japan; 3 Department of Radiology, Hyogo Ion Beam Medical Center, 1-2-1 Kouto, Shingu-cho, Tatsuno, Hyogo, 679-5165, Japan; 4 Division of Physics, Electrical and Computer Engineering, School of Engineering, Yokohama National University, 79-5 Tokiwadai, Hodogaya-ku, Yokohama, 240-8501, Japan

**Keywords:** Carotid artery deformation, Maxillofacial catheter navigation, Computed tomography angiography

## Abstract

**Background:**

To improve the accuracy of catheter navigation, it is important to develop a method to predict shifts of carotid artery (CA) bifurcations caused by intraoperative deformation. An important factor affecting the accuracy of electromagnetic maxillofacial catheter navigation systems is CA deformations. We aimed to assess CA deformation in different head and neck positions.

**Methods:**

Using two sets of computed tomography angiography (CTA) images of six patients, displacements of the skull (maxillofacial segments), C1–C4 cervical vertebrae, mandible (mandibular segment), and CA along with its branches were analyzed. Segmented rigid bones around CA were considered the main causes of CA deformation. After superimposition of maxillofacial segments, C1–C4 and mandible segments were superimposed separately for displacement measurements. Five bifurcation points (vA–vE) were assessed after extracting the CA centerline. A new standardized coordinate system, regardless of patient-specific scanning positions, was employed. It was created using the principal axes of inertia of the maxillofacial bone segments of patients. Position and orientation parameters were transferred to this coordinate system. CA deformation in different head and neck positions was assessed.

**Results:**

Absolute shifts in the center of gravity in the bone models for different segments were C1, 1.02 ± 0.9; C2, 2.18 ± 1.81; C3, 4.25 ± 3.85; C4, 5.90 ± 5.14; and mandible, 1.75 ± 2.76 mm. Shifts of CA bifurcations were vA, 5.52 ± 4.12; vB, 4.02 ± 3.27; vC, 4.39 ± 2.42; vD, 4.48 ± 1.88; and vE, 2.47 ± 1.32. Displacements, position changes, and orientation changes of C1–C4 segments as well as the displacements of all CA bifurcation points were similar in individual patients.

**Conclusions:**

CA deformation was objectively proven as an important factor contributing to errors in maxillofacial navigation. Our study results suggest that small movements of the bones around CA can result in small CA deformations. Although patients’ faces were not fixed properly during CT scanning, C1–C4 and vA–vE displacements were similar in individual patients. We proposed a novel method for accumulation of the displacement data, and this study indicated the importance of surrounding bone displacements in predicting CA bifurcation.

## Background

Superselective intra-arterial chemoradiotherapy has a potent anticancer effect and is feasible for the treatment of head and neck cancer [1-4]. In this method, the tip of a catheter must be inserted into a target artery through the superficial temporal artery (STA) or femoral artery to deliver the anticancer drugs. In general, surgeons or interventional radiologists identify the position of the catheter tip intra-operatively and target arteries under fluoroscopic guidance. However, there are problems associated with this technique such as the technical difficulty of catheterization under two-dimensional (2D) fluoroscopic imaging, the increased use of contrast medium and radiation exposure, and the long operation time. Therefore, we developed a novel electromagnetic (EM) maxillofacial catheter navigation system using three-dimensional (3D) guidance to resolve these problems [5].

Preoperative imaging such as computed tomography (CT), magnetic resonance imaging (MRI), and X-ray fluoroscopy are required for registration in patients undergoing navigation. Several methods such as point-based registration using skin markers [6-8], surface-based registration [9,10] matched at many points of the facial surface, and 2D–3D registration [11] mapping intraoperative 2D radiographic images on preoperative 3D modeling images, have been reported. A rigid, custom-made mouthpiece with fiducial markers has also been used [12] to prevent errors because of facial skin shift [13]. Furthermore, laser scan-based registration, in which the surface shape of a patient (for example their facial surface) is measured and registered to the model has better registration accuracy, because a greater number of points on the facial surface are used for registration [14]. One of the most important processes for surgical navigation using optical navigation systems is the “registration process.” When using a catheter navigation system in the head and neck region, the deformation of the carotid artery (CA), which forms the path of the guide wire and the catheter attached to the EM sensor, must be considered an important factor affecting accuracy because the artery is not a rigid body like bone and instead consists of soft tissue. Therefore, displacement of the CA bifurcation points in different head and neck positions need to be clarified.

Retrograde superselective intra-arterial catheterization for advanced oral cancer is commonly performed above the carotid bifurcation, and the catheter tip is inserted into branches of the external carotid artery (ECA) such as the maxillary artery (MA), facial artery (FA), and lingual artery (LA). In this method of catheterization, it is most important to identify the 3D positions of the branches of the ECA rather than the distal points of target arteries. The CA may be deformed extensively by movement of the patient’s head, neck, and shoulder. On the other hand, the CA is located in the central area of the neck. The arterial wall is thicker than the vein wall; hence, its deformation is less than that of veins. From an anatomical viewpoint, changes in head and neck position (rotation, anteflexion, and retroflexion) and mouth opening/closing can be expressed as movement of a series of cervical vertebrae (C1–C7) as well as the mandible. In addition, the deformation of the surrounding soft tissues such as the sternocleidomastoid muscle and the internal jugular vein are also considered to affect deformation of the CA. Because deformation of surrounding soft tissues is also affected by the displacement of surrounding bone segments, the movement of rigid bodies around the CA was assumed to be the main causes of CA deformation in this study. We analyzed the patient’s computed tomography angiography (CTA) images since the image data include both vascular shape and surrounding bone segments. Thus, the 3D displacements of CA bifurcation points and the displacement (position and orientation) of surrounding bone segments were analyzed.

## Methods

### Patient selection

CTA image data of advanced cancer patients were obtained twice for each patient once before and once after superselective intra-arterial chemoradiotherapy. The analysis included the angiographic data of the non-catheterized CAs (left or right) of all patients. Of the oral cancer patients in whom the right or left CA was not catheterized, 6 patients were selected at random [male: female = 5:1, age: 39–70 (mean: 59 years)]. The interval between acquisition of the two images was 82–95 days (mean: 89 days). Three left carotid arteries and three right ones were assessed in this study. According to laser alignment of the CT scanner, the Frankfort horizontal plane of the patients was perpendicular to the table, allowing for the determination of the midline of the face. Then, the forehead and chin of the patients were fixed using belts. Patients’ faces were not fixed properly; therefore, the head and neck positions of the patients were different to some extent while performing CTA.

### Image data acquisition

A spiral CT scanner (Aquilion 64; Toshiba Medical Systems, Tokyo, Japan) with 0.5-mm × 64-slice collimation was used. Scanning conditions were 120 kV, 250 mA, slice interval: 0.5 mm, slice thickness: 1.0 mm, 512 × 512 pixel, pixel size: 0.43 × 0.43 mm^2^. Patients were instructed to stop breathing and avoid swallowing during scanning. A non-ionic contrast medium (100 mL) was injected at a rate of 4.0 mL/s through an antebrachial vein. The region of interest (ROI) was set in the common CA (CCA) to measure the arrival time of the bolus. The scanning procedure started automatically when an enhancement level of 90 Hounsfield Units was reached in the ROI. CTA image data and non-enhanced CT image data were acquired as Digital Imaging and Communication in Medicine (DICOM) data. The study was approved by the Institutional Review Board of the Yokohama City University Graduate School of Medicine.

### Data analysis

**Figure 1:**
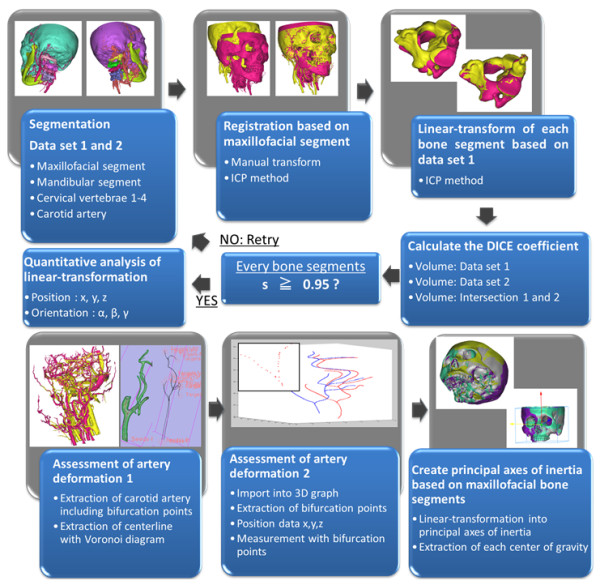
Outline of carotid artery deformation analysis.

An outline of our data analysis is shown in Figure 1:

i. Selection of bone segments and carotid arteries to be analyzed

The maxillofacial segment was considered as the fiducial bone segment. The mandibular condyle leads the anterior sliding movement as well as the hinge movement during mouth opening/closing. The FA and its branches are close to the inferior border of the mandible and run across it. The MA is close to the condylar neck of the mandible and runs medially. The position of the LA also relates to mandibular movement. Therefore, the position of these arteries, which are target arteries in superselective intra-arterial infusion for oral cancer, can change according to mandibular position. The cervical vertebrae (C1–C7) support the head. The CA runs close to the cervical vertebrae. The atlantoaxial joint, which can affect head motion such as rotation, is located between C1 and C2. Furthermore, C1–C4 are close to the CA bifurcation, the internal carotid artery (ICA), and the external carotid artery (ECA) and its branches. Based on these anatomical considerations, the movement (translation and rotation) of C1–C4 was assumed to be the main factor determining CA deformation.

**Figure 2:**
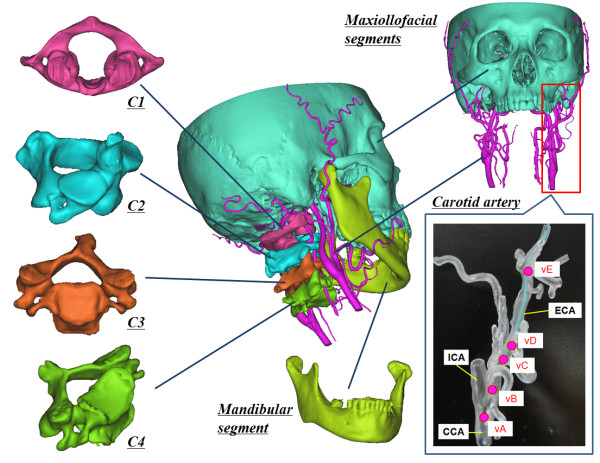
Surface rendering models.

The segmented bone fragment models and CA models were as follows (Figure 2):

Skull (maxillofacial segments)

First cervical vertebra (atlas, C1)

Second cervical vertebra (axis, C2)

Third cervical vertebra (C3)

Fourth cervical vertebra (C4)

Mandible (mandibular segment)

Carotid artery (CCA, ECA, ICA, LA, FA, MA, occipital artery (OA), STA)

ii. Segmentation with identity evaluation of generated models based on the DICE coefficient

Pre- and post-treatment CTA images (data set 1 and data set 2) were segmented using commercially available image-processing software (Mimics ver.14.01; Materialize, Leuven, Belgium).

First, the threshold of Hounsfield units required to distinguish ROIs from non-ROIs in CTA image data set was determined. All ROIs corresponded to bones and vasculature (“a”) in the head and neck. Second, the threshold of double scanned non-enhanced CT images was determined and ROIs were determined. These ROIs were the only bone segments (“b”) without vasculature in the head and neck. Two surface rendering models from each image data set were reconstructed and the registration process was performed using an iterative closest point (ICP) method. Third, “a” was subtracted from “b” to segment the vascular model by a Boolean operation method. Fourth, the model reconstructed from the CTA image data set was subtracted from the vascular model. Fifth, the mandible was segmented. After the mandibular region was completely separated from the other regions by a manual deletion procedure, the mandibular region was reconstructed using the region growing method. The region growing method makes it possible to split the segmentation created by the thresholds of several objects and to remove the discontinuous regions near the surrounding voxels. Finally, segmentation procedures of C1 to C4 vertebrae were performed by repetition of the above procedure. In conclusion, two sets of surface rendering models (C1–C4, mandible, and CA in data sets 1 and 2) were created in all patients.

The data regarding all bone and vascular models were obtained in the form of STL (Standard Triangulated Language) files. All models created in data set 2 were imported into data set 1. And, the models of data set 2 were superimposed into that of data set 1 on the basis of each maxillofacial model by an automated ICP method. Then the transformation matrix T for this displacement was calculated.

Next, each C1–C4, mandible, and CA model of data set 2 was superimposed on that of data set 1 by ICP method. Then, the transformation matrices T_C1_, T_C2_, T_C3_, T_C4_, T_mandible_, and T_CA_ that superimpose these models were calculated. There are always measurement errors caused by the segmentation and registration process as well as by CT scanning. In particular, the segmentation procedure results in several errors. The representative causes of errors are the procedure for manual deletion of the continuous parts among the bone segments and between the bone and vasculature segments, the deletion procedure of metal artifacts, and time-dependent distribution changes in the CT values in bone segments. Thus, the DICE coefficient *s* was introduced to measure the similarity of segmentation.

A pair of bone models for the same segments in pre- and post- data set was aligned to each other using ICP algorithm. The volumes of the corresponding models of each bone segment (either of the maxillofacial bone, C1–C4 or the mandible) in both data sets 1 and 2 were calculated. Since the same bone segment from the respective CT images is extracted, the two volumes should have similar values if segmentation is adequate. Thus, the segmentation process was repeated until the value of *s* was greater than 0.95.

iii. Assessment of the displacement of each bone segment between two data sets

The displacements of the bone segments other than the maxillofacial segment were measured when the bone models of data set 2 were superimposed on that of the data set 1 using the transformation matrices T_C1_, T_C2_, T_C3,_ T_C4_, and T_mandible_. Each vector of x, y, and z axis in the world coordinate system was roughly in the posterior, left, and superior direction of the patients. For example, when the bone model was moved to the posterior by 1 mm, left by 2 mm, and superiorly by 3 mm, the x, y, z position coordinates were x,1; y, 2; z, 3; respectively. In the orientation coordinates, each rotation angle was calculated around each x, y, z axis. These rotation angles were regarded as α, β, and γ (Euler Angle), respectively. For example, when the model was rotated 1° around the central x axis, 2° around the central y axis, and 3° around the central z axis, the x, y, z orientation coordinates were α,1; β, 2; γ, 3 (rotation order: α → β → γ), respectively.

These displacements were measured in the pre-treatment CTA coordinate system. Then, a novel standardized coordinate system was defined as the coordinate system created using the principal axes of inertia of the maxillofacial bone segments in patients. All displacements (translation and rotation of C1–C4 vertebrae and mandible as well as the translation of CA bifurcations) mentioned later are determined on the basis of the standardized coordinate system.

iv. Assessment of CA bifurcation displacement

The centerlines of CA including its branches were extracted with the voronoi diagram method (3D slicer ver.3.6.3, http://www.slicer.org). Next, the position coordinates x, y, z of all the points in the extracted centerlines were extracted and displayed on a 3D graph. The position coordinates of CA bifurcations which are important in the catheter navigation for oral cancer were manually obtained from the 3D graph. The following five bifurcation points were analyzed in the present study:

vA: bifurcation between ICA and ECA. vB: bifurcation between LA and ECA.

vC: bifurcation between FA and ECA. vD: bifurcation between OA and ECA.

vE: bifurcation between MA and ECA.

3D displacement of all the bifurcation points was calculated in the coordinate system of data set 1.

v. Assessment of the displacement data among several patients

We obtained the 3D displacement data of 5 bone segments (mandible and C1–C4) and 5 CA bifurcation points relative to the maxillofacial bone segment for each patient’s data sets. The X, Y, Z system of coordinates used in the data representation was arbitrarily defined dependent on CT data acquisition for the initial imaging process. To compare the displacement data among several patients, a standardized coordinate system based on anatomical landmarks should be defined and all the data should be represented using this coordinate system. We set the principal axes of inertia of the maxillofacial bone segment as the three axes of the standard coordinate system. The origin of the coordinate system was set at the center of gravity of the maxillofacial bone segment model in each patient. The center of gravity and the principal axes of inertia in the maxillofacial bone model were calculated using commercially available software (3 matic ver.6.0, Materialize, Leuven, Belgium). Each vector of the x, y, z axis led roughly to the posterior, left, and superior direction of the maxillofacial segment. By transforming all the displacement data for bone segments and CA bifurcations of each patient into the standardized coordinate systems based on the maxillofacial bone segment of each patient, the displacement data of all the patients can be discussed using an anatomically standardized coordinate system independent of CTA image data acquisition conditions.

**Figure 3:**
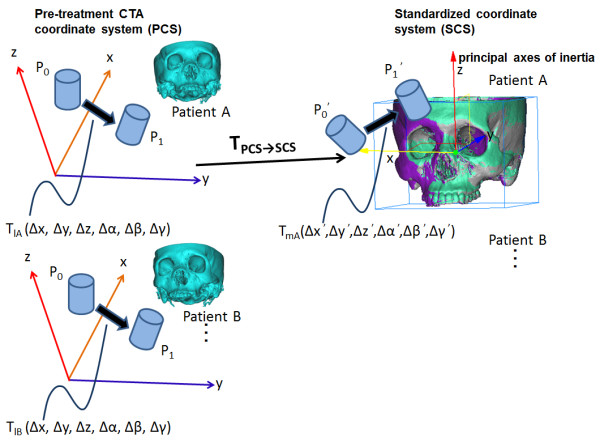
**Accumulation of the displacement data among several patients.** “Pre-treatment CTA coordinate system (PCS)” was set in data set 1. P_0_ and P_1_ stand for the models of the bones or bifurcation points of CA. The position and orientation parameter T_l_ for moving from P_0_ to P_1_ was obtained in PCS. The transformation matrix T_PCS→SCS_ was calculated to create the standardized coordinate system (SCS) from the principal axes of inertia of the maxillofacial model. Here, T_mA,B_… = T_PCS→SCS_ T_lA,B_…T_PCS→SCS_^−1^. The calculated T_mA,B_… represents the position and orientation of bone and artery models when T_lA,B_… was transformed to SCS.

Δx, Δy, Δz, Δα, Δβ, and Δγ indicate the changes in position and orientation of some bone models in the pre-treatment CTA coordinate system. Consequently, Δx´, Δy´, Δz´, Δα´, Δβ´, and Δγ´ were regarded as the new parameters mapped in the standardized coordinate system. The relationship of the two coordinate systems is represented in Figure 3.

## Results

In this study, segmentation of all bone models was performed with high quality (*s* range; 0.96–1.00). The absolute shifts of the centers of gravity of C1–C4, mandible, and 5 bifurcation points are shown in Table 1. The shifts in each bone model were indicated by mean ± standard deviations and maximum values as follows: C1, 1.02 ± 0.9 mm (max 2.19); C2, 2.18 ± 1.81 mm (max 4.68); C3, 4.25 ± 3.85 mm (max 9.83); C4, 5.90 ± 5.14 mm (max 13.38); mandible, 1.75 ± 2.76 mm (max 7.34). The shifts in the CA bifurcations were as follows: vA, 5.52 ± 4.12 mm (max 12.49); vB, 4.02 ± 3.27 mm (max 10.04); vC, 4.39 ± 2.42 mm (max 9.18); vD, 4.48 ± 1.88 mm (max 8.03); vE, 2.47 ± 1.32 mm (max 4.59).

**Table 1:** Absolute shifts of center of gravity in each bone model and carotid artery bifurcation points

**No.**	**C1**	**C2**	**C3**	**C4**	**mandi**	**vA**	**vB**	**vC**	**vD**	**vE**
#1(L)	1.99	4.18	8.31	11.43	0.95	6.78	3.75	3.07	4.62	2.36
#2(L)	0.13	0.40	0.72	1.39	7.34	1.43	1.94	3.13	4.23	2.04
#3(L)	0.32	0.96	1.86	3.03	0.50	3.54	2.50	3.43	2.56	2.91
#4(R)	1.16	1.88	3.17	3.91	1.01	6.86	4.96	4.52	3.93	2.37
#5(R)	2.19	4.68	9.83	13.38	0.48	12.49	10.04	9.18	8.03	4.59
#6(R)	0.35	0.97	1.61	2.26	0.20	2.03	0.91	2.99	3.51	0.53

(*L*): left carotid artery, (*R*): right carotid artery, mandi: mandible, *vA*: bifurcation between ICA and ECA, *vB*: bifurcation between LA and ECA, *vC*: bifurcation between FA and ECA, *vD*: bifurcation between OA and ECA, *vE*: bifurcation between MA and ECA. Cervical vertebrae 1–4 are indicated by C1–C4, respectively.

**Figure 4:**
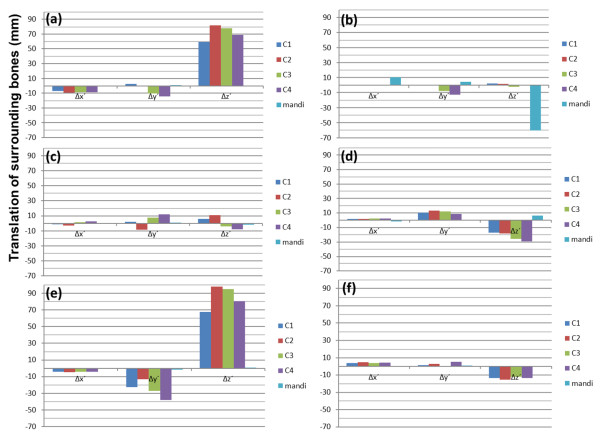
**The position parameters of surrounding bones (Δx´, Δy´, Δz´).** The parameters (Δx´, Δy´, Δz´) were the results when the position parameters (Δx, Δy, Δz) of bones (C1–C4 and mandible) were transformed to the parameters in the standardized coordinate system. X axes define the parameters of each bone segment. Y axes define the translation of surrounding bones in the standardized coordinate system. Patients 1–6 correspond to graphs (**a**)–(**f**) in Figures 4, 5, 6. Values are in mm.

**Figure 5:**
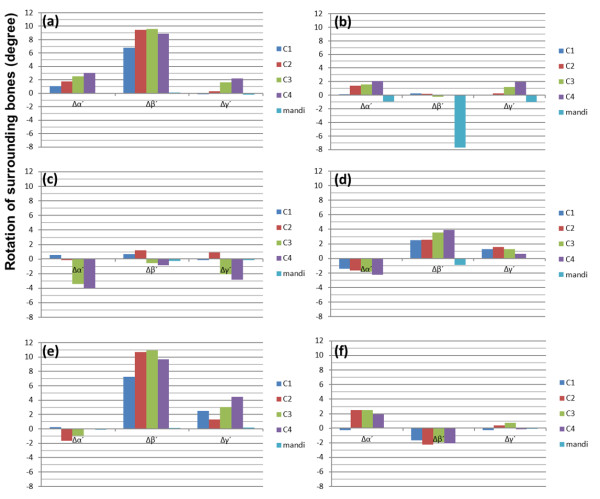
**The orientation parameters of surrounding bones (Δα´, Δβ´, Δγ´).** The parameters (Δα´, Δβ´, Δγ´) were the orientation parameters (Δα, Δβ, Δγ) of bones (C1–C4 and mandible) were transformed to the parameters in the standardized coordinate system. X axes define the parameters of each bone segment. Y axes define the rotation of surrounding bones in the standardized coordinate system. Patients 1–6 correspond to graphs (**a**)–(**f**) in Figures 4, 5, 6. Values are in degree.

**Figure 6:**
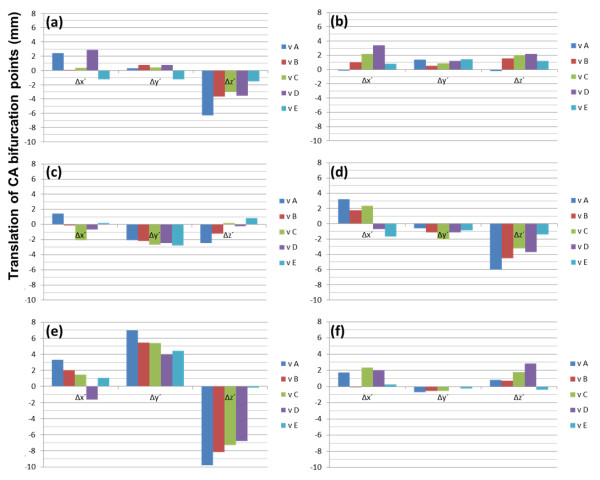
**The position parameters of carotid artery bifurcations (Δx´, Δy´, Δz´).** The parameters (Δx´, Δy´, Δz´) were obtained when the position parameters (Δx, Δy, Δz) of the bifurcations of CA were transformed to the parameters in the standardized coordinate system. The bifurcations of the CA were assessed at each bifurcation point. These points were not contained in the orientation parameters. X axes define the parameters of each CA bifurcation points. Y axes define the translation of CA bifurcations in the standardized coordinate system. Patients 1–6 correspond to graphs (**a**)–(**f**) in Figures 4, 5, 6. Values are in mm.

Translations of C1–C4 and mandible are shown in Figure 4 for 6 patients. Rotations of C1–C4 and mandible are displayed in Figure 5. Translations of CA bifurcation, vA–vE, are also shown in Figure 6. Displacements, position changes, and orientation changes of C1–C4 segments as well as the displacements of all CA bifurcation points were similar in individual patients.

## Discussion

In order to assess 3D movement of bone segments between two different sets of volumetric data under separate conditions, accurate registration of the identical bone segments is required. In this study, we measured the DICE coefficient *s* using the overlapping volume of the segmented models as an index of segmentation quality. Since *s* was larger than 0.96, it is considered that the bone segments used in this study were segmented accurately from each set of data from the 6 patients. We believe that CA was extracted sufficiently because the CA contrast was clearly visible on the CTA image. We judged the anatomical validity of CA bifurcation points by referring to the contour of vasculature. However, these assessments could have measurement errors. In future studies, the accuracy and variability of segmentation of CA and identification of the bifurcations should be assessed so as to support the data obtained in this study more reliably.

The position and orientation parameters in CT volumetric data were represented in the initial DICOM coordinates (pre-treatment CTA coordinate system). Thus, the data are influenced by the relative position of the patient in the CT coordinate system. To accumulate displacements data under different imaging conditions, the obtained data should be transferred to the standardized coordinate system. Therefore, we set the coordinate system determined by the principal axes of inertia in the individual maxillofacial models.

CA deformation is obviously caused by changes in the head and neck position. In the present study, CA deformation was actually assessed using CTA data scanned in the supine position before and after treatment of oral cancer. Some CA bifurcation points shifted by more than 1 cm. This implies that preoperative data-based catheter navigation can include substantial positioning errors because of CA deformation. Additionally, in discussions on the accuracy of EM tracking systems [15,16], CA deformation was considered to be more important than the registration or calibration procedure as a factor affecting errors in catheter navigation. In this study, the range of CA deformation was assessed at 5 important CA bifurcations for catheterization. When patients 2, 3, and 6 with small centroid shifts in the cervical vertebrae were defined as group A, and patients 1, 4, and 5 with large centroid shifts of the cervical vertebrae were defined as group B, the average centroid shifts of the C1–C4 models in groups A and B were 1.17 mm and 5.51 mm, respectively. As a result, the average shifts of CA bifurcation in group A and B were 2.51 mm and 5.84 mm, respectively. CA deformation was considered to be small when centroid movement in the surrounding bone models was small. The influence of mandibular displacement on CA deformation was not clear, because there were no significant mandibular displacements in 5 patients because of a stable occlusal position.

The correlation between the displacements of the surrounding bones and CA deformation was analyzed. C4 is most distal from the maxillofacial bone. C2 is mainly moved by motion of the atlantoaxial joint during rotation of the neck. Thus, C4 and C2 were selected for analysis. To represent rotation and anteflexion of the head, differences between translations and rotations of C4 and C2 were used. The following parameters were calculated: *x*_1_ (C4Δx´ − C2Δx´), *x*_2_ (C4Δy´ − C2Δy´), *x*_3_ (C4Δz´ − C2Δz´), and *x*_4_ (C4Δα´ − C2Δα´) and selected as 4 parameters representing the displacements of surrounding bones caused by head rotation and anteflexion. These 4 parameters were fitted to the displacement of CA bifurcation points by linear multiple regression analysis.

That is,

(1) $$\begin{array}{l} {\hat{y}}_{i,j}=b_{0}+{\text{b}}_{1}x_{1}+b_{2}x_{2}+{\text{b}}_{3}x_{3}+b_{4}x_{4}\\ \left(\text{i},=\text{vA},\text{vB}\ldots \text{vE},\text{j}=\Delta \text{x}\prime ,\Delta \text{y}\prime ,\Delta \text{z}\prime \right)\\ x_{1}\left(\text{C}4\Delta \text{x}\prime -\text{C}2\Delta \text{x}\prime \right),\;x_{2}\left(\text{C}4\Delta \text{y}\prime -\text{C}2\Delta \text{y}\prime \right),x_{3}\left(\text{C}4\Delta \text{z}\prime -\text{C}2\Delta \text{z}\prime \right),\text{and}x_{4}\left(\text{C}4\Delta \alpha \prime -\text{C}2\Delta \alpha \prime \right) \end{array}$$

**Figure 7:**
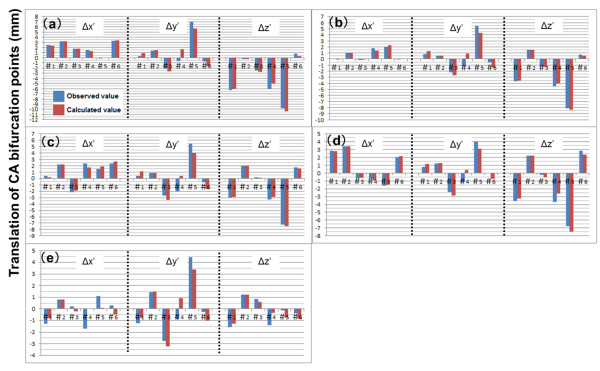
**Differences between the observed and calculated variables.** Differences between the observed and calculated variables by multiple-linear regression equations using position parameters of each CA bifurcation are shown in this graph. (**a**)–(**e**) represent vA–vE, respectively. There are three sets (Δx´, Δy´, Δz´) of data for patients 1–6 (#1–#6) shown on the X axes of the graphs. Y axes define the translation of CA bifurcation points (mm).

*b*_*0*_ was a constant and *b*_*1*_, *b*_*2*_, *b*_*3*_, *b*_*4*_ were partial regression coefficients. The relationship between the calculated values using the multiple regression model and the observed values is indicated in Figure 7. We suggest the possibility that the displacement (position and orientation) of surrounding bones is related to the displacement (position) of CA bifurcations. Although angiographic data of patients’ non-catheterized CAs (left or right) are rare because of the clinical protocol used for CA catheterization, analysis data for a larger number of cases will be required to confirm this hypothesis.

For the clinical application of catheter navigation, a method to minimize the shift in the pre- and intra-operative positions of the mandible and of the cervical vertebrae is required. To prevent this shift, a facial mask can be used as a non-invasive immobilization system [17,18]. Furthermore, invasive methods are available to fix the head using head pins. The shift in the CA bifurcation was approximately 2–3 mm without large movement of the cervical vertebrae and mandible although this result should have measurement errors.

To improve accuracy of catheter navigation, it is important to develop a method to predict shifts in CA bifurcations because of intraoperative deformation. The present study indicated the importance of the movement of surrounding bones in predicting CA bifurcation. The position and orientation of the surrounding bones can be measured using intraoperative X-ray images, and the intraoperative position and orientation can be compared with the preoperative ones. By investigating the correlation between the displacements of surrounding bones and CA bifurcation using accumulated clinical data, we will be able to develop a method to predict CA bifurcation points without a contrast medium for fluoroscopic images.

In addition, a registration method by which the path of a sensor attached to an intravascular catheter corresponds to the artery centerline in CTA images is required. The registration method between the intravascular path and the vessel centerline in the preoperative images has been reported in cerebrovascular surgery [19], bronchoscopy [20], and cardiovascular surgery [21]. Our research group has also reported its possible application in the head and neck [5]. In future studies, the results of the present study will provide the reference data.

## Conclusions

CA deformation was considered to be small according to the small displacements (translation and rotation) of the cervical vertebrae. In cases with substantial rotation and anteflexion of the head, the displacement of CA bifurcation points was as large as 5.84 mm. This implies that preoperative data-based catheter navigation can include substantial positioning errors because of CA deformation. Displacement (translation and rotation) of the cervical vertebrae (C1–C4) was analyzed by comparing two sets of CTA data scanned before and after chemoradiotherapy. The translation of CA bifurcation points was also analyzed. All analyses were conducted using a standardized coordinate system independent of CTA image data acquisition conditions.

In the present study, when the patients underwent CT, their head was not fixed properly. Under these conditions, the displacements, changes in positions, and orientations of C1–C4 segments were similar in all patients. The displacements of CA bifurcation points vA–vE were also similar in all patients. We proposed a novel method for accumulation of the displacement data, and this study indicated the importance of surrounding bone displacements in predicting CA bifurcation.

## Abbreviations

3D: Three-dimensional; EM: Electromagnetic; CT: Computed tomography; MRI: Magnetic resonance imaging; CA: Carotid artery; CTA: Computed tomographic angiography; ROI: Region of interest; CCA: Common carotid artery; C1: First cervical vertebra; C2: Second cervical vertebra; C3: Third cervical vertebra; C4: Fourth cervical vertebra; ECA: External carotid artery; ICA: Internal carotid artery; LA: Lingual artery; FA: Facial artery; MA: Maxillary artery; OA: Occipital artery; STA: Superficial temporal artery; ICP: Iteractive closest point; DICOM: Digital imaging and communication in medicine; STL: Standard triangulated language; vA: Bifurcation of ICA and ECA through CCA; vB: Bifurcation of LA through ECA; vC: Bifurcation of FA through ECA; vD: Bifurcation of OA through ECA; vE: Bifurcation of MA through ECA.

## Competing interests

The authors declare that they have no competing interests. The authors alone are responsible for the content and writing of the paper.

## Authors’ contributions

All the authors contributed to the preparation of the manuscript and then approved its final version.
